# Inhaled Loxapine for the Treatment of Psychiatric Agitation in the Prehospital Setting: A Case Series

**DOI:** 10.5811/cpcem.2017.5.33840

**Published:** 2017-10-06

**Authors:** Armando Cester-Martínez, José A. Cortés-Ramas, Diego Borraz-Clares, Marta Pellicer-Gayarre

**Affiliations:** Zaragoza Fire Department, Emergency Medical Services, Zaragoza, Spain

## Abstract

Rapid and effective control of agitated patients is crucial for ensuring their safety and proper management. We present a case series of 12 agitated psychiatric patients who were suitable for treatment with inhaled loxapine in the prehospital emergency setting. Two refused its administration and two required additional treatment. Loxapine was effective within 2–10 minutes, with no adverse effects or sedation. In our experience the use of inhaled loxapine enabled rapid and non-coercive control of agitation in most psychiatric patients, allowing us to avoid mechanical restraint and injectable drugs, and facilitating the transportation and transfer of the patients.

## INTRODUCTION

The incidence of psychiatric agitation in a prehospital setting has been estimated to account for around 2–4% of all emergency calls,^1^ although these figures may not reflect its actual frequency. Agitation may be underdiagnosed due to methodological issues, given that different criteria are used to identify “agitated patients”, and substantial differences exist among emergency medical services (EMS).^1^–^4^ Thus, it is not uncommon for paramedics and emergency medical staff to deal with agitated and uncooperative patients – not an easy task as they are working under great pressure and managing different medical conditions. The potential risk of agitated patients escalating to aggressive and violent behavior puts patients, staff and crew at risk. Therefore, rapid control of these episodes becomes crucial in this scenario.^2^–^6^

The options for managing psychiatric agitation are limited and include verbal de-escalation, pharmacologic control (“rapid tranquilization”) and physical restraints.^3^–^8^ Although various drugs can be given to control agitation, there is no consensus on which is the best option to manage agitation in the prehospital setting. The route of administration and how to determine which patients require sedation have also been the subject of debate.^6^, ^7^ In this regard, in 2012 a workgroup of the American Association for Emergency Psychiatry issued a consensus statement about Project BETA, the Best Practices in Evaluation and Treatment of Agitation in the emergency department.^5^, ^9^ This consensus discussed the best-practice pharmacologic approaches to use when agitation requires emergency management before stabilizing the underlying etiology. Among them is a new formulation of a previously extensively marketed antipsychotic, inhaled loxapine, considered a good option in cooperative agitated patients in some cases.^5^ The efficacy and safety of inhaled loxapine has been demonstrated in the emergency and hospital setting,^10^ although experience in the prehospital field is limited.

## CASE REPORT SERIES

We present a case series of 12 agitated psychiatric patients who were suitable for treatment with inhaled loxapine in the prehospital setting by the Zaragoza (Spain) Fire Department EMS from December 2015 to July 2016. Our EMS covers a catchment area of around 700,000 inhabitants. Patients are attended by a team comprising a physician, a nurse and a paramedic.

Inhaled loxapine was used in patients with agitation related to schizophrenia, bipolar disorder or schizoaffective disorder. Patients with agitation not related to psychotic disease or with clinically significant acute or chronic pulmonary disease were not treated with inhaled loxapine.

The decision to treat each patient was based on the discretion of the attending physician. The psychiatric diagnosis was either reported by the family or caregiver, by medical discharge report, or already recorded in our files of previously known patients. Clinical diagnosis of agitation due to psychotic disease, absence of respiratory symptoms and absence of overt drug intoxication were confirmed during the verbal de-escalation procedure.

Agitation intensity was assessed on-site with the *Clinical Global Impression–Severity scale* (CGI–S),^11^ and with the *Positive and Negative Syndrome Scale–Excited Component* (PANSS–EC)^12^ during the debriefing of each case, back at the EMS base. The PANSS-EC evaluates acute agitation in psychiatric patients and consists of five items: excitement, tension, hostility, uncooperativeness, and poor impulse control. Each item is rated from 1 (not present) to 7 (extremely severe) and total PANSS-EC scores range from 5 to 35; scores above 25–30 correspond to severe agitation in clinical practice. The CGI-Severity scale (CGI-S) asks the clinician one question: “Considering your total clinical experience with this particular population, how mentally ill is the patient at this time?” which is rated on the following seven-point scale: 1=normal, not at all ill; 2=borderline mentally ill; 3=mildly ill; 4=moderately ill; 5=markedly ill; 6=severely ill; 7=among the most extremely ill patients.

Patient characteristics and treatment responses are shown in table. Regarding the severity of agitation, two patients were classified as “mild,” two as “moderate,” two as “moderately severe,” three as “severe” and, finally, three patients presented with “extreme agitation.” After initial verbal de-escalation was performed in all patients, inhaled loxapine was offered and administered to all patients except for two, both rated as “extremely agitated,” who required mechanical restraint and additional treatment with intranasal midazolam (10 mg) and intravenous haloperidol (10 mg). Most patients (8/10, 80%) received just one dose of inhaled loxapine, which was effective within minutes after administration (mean: 6 minutes, range: 2–10 minutes). Two patients required additional medication (intranasal midazolam) to control agitation. All patients were safely transported and transferred to the hospital within 30–45 minutes. No clinically significant adverse events were observed.

## DISCUSSION

To our knowledge, this case series represents the first report of inhaled loxapine use in agitated patients in the prehospital setting. In our experience, inhaled loxapine was rapid, effective, well-tolerated and accepted by most patients, even in those severely agitated.

Our main aim in agitated patients is to ensure their safety and to control symptoms immediately, in order to assess and manage any risk to life and transport them to the hospital. The potential for escalation into aggressive and violent behavior makes it imperative to address agitated patients rapidly and efficiently; however, following current recommendations we try to avoid the strategy of “restraint and sedate,” as this practice is far from optimal.^3^–^5^, ^13^, ^14^ In the hospital setting, a minimum of five health professionals is recommended for performing an appropriate mechanical restraint, which is not feasible in a prehospital setting.^13^, ^14^ Moreover, potential serious adverse events such as rhabdomyolysis, acidosis and sudden death have been associated with physical restraints.^4^, ^7^, ^13^ Finally, coercive interventions are traumatic and could impair the physician-patient relationship and effective longer-term management after resolving the agitation episode.^2^, ^5^, ^13^

**CPC-EM Capsule**

What do we already know about this clinical entity?

Agitated patients are seen frequently in emergency medical systems, and the risk of agitation escalating to aggressive and violent behavior demands rapid control of these patients.

What makes this presentation of disease reportable?

*This is the first report of an* *emergency medical services* *clinical experience using inhaled loxapine in agitated patients in a prehospital setting.*

What is the major learning point?

Inhaled loxapine allows a rapid and non-coercive control of agitation in certain psychiatric patients.

How might this improve emergency medicine practice?

In prehospital settings, the use of inhaled loxapine can replace the use of mechanical restraint and injectable drugs, thus facilitating patient transfer to the hospital.

After initial verbal de-escalation, we were able to convince all but two patients to use inhaled loxapine. The two patients who refused treatment were extremely agitated, and it was not possible to avoid restraining them. However, one additional patient who was in the same extreme state was persuaded through verbal de-escalation to use inhaled loxapine. In that case, as in another severely agitated patient, the rapid partial effect of inhaled loxapine allowed us to administer other non-coercive, more sedative medication, such as intranasal midazolam, without restraining them. This reinforces the notion that using non-invasive formulations improves the overall patient experience and furthers cooperation between patients and healthcare providers.

In relation to pharmacologic management, current prehospital treatments are based on the ED’s practices, although sedating agitated patients in the ED differs significantly from sedation in a prehospital environment.^6^, ^7^, ^15^, ^16^ Parenteral benzodiazepines, and first- and second-generation antipsychotics, alone or in combination, are primarily used because of their sedative effects and rapid onset of action. Intramuscular (IM) ketamine and intranasal midazolam have also been used as good sedative options.^16^, ^17^ Antipsychotics should be considered first-line treatment in psychotic agitated patients because they address the underlying disease.^4^, ^5^, ^8^, ^13^, ^16^

Until recently, loxapine as an IM formulation has been widely used in EDs in Canada and France to control agitation.^18^ Inhaled loxapine was approved by the FDA in 2012 and the European Medicines Agency in 2013, and is available in the U.S. and most European countries. The new inhaled formulation delivers loxapine as fast as an intravenous injection, and has demonstrated onset of action within 10 minutes of administration.^5^, ^10^, ^13^, ^17^, ^18^ In fact, we observed a rapid and effective response in eight of 10 patients, in some cases occurring well before 10 minutes had passed since administration, in agreement with what has been published in other case series.^19^, ^20^

In our patients no adverse reactions occurred and, significantly, we observed no over-sedation. Patients who received inhaled loxapine were easily transported and transferred, calm and awake, to the hospital in a suitable state for a formal psychiatric evaluation and proper treatment. Using inhaled loxapine could result in an improvement of the patient’s subsequent clinical management and shorten his length of stay in the ED, alleviating the burden on prehospital and ED staff.

Finally, it is worth noting that inhaled loxapine was not accepted by two patients, both classified as extremely agitated. It should be taken into account that inhaled loxapine is self-administered under medical supervision, and a minimal cooperation from patients is required. This medication is not suitable in situations where verbal de-escalation is not successful and patients are actively refusing treatment. More restrictive management must be followed in these cases.^4^, ^13^

While this case series reports a new approach for the immediate treatment of agitated patients in the prehospital setting, some limitations should be discussed. First, the lack of an active control did not allow for any direct comparison with existing treatments for agitation. And because psychiatric diagnoses were based mainly on family reports and through our clinical assessment during the verbal de-escalation procedure, other psychiatric comorbidities could not be ruled out. Finally, there was no patient follow-up following hospital transfer. Therefore, the absence of intoxication was not confirmed by tests and thus was not assessed.

## CONCLUSION

Despite the limitations noted above, these initial case reports in a prehospital setting indicate that inhaled loxapine may represent an improvement in the management of certain agitated patients in this setting. Therefore, self-harm and associated problems may be considerably reduced. Future studies with a larger number of subjects and comparison with injectable as well as oral medications to control agitation are needed to corroborate these benefits.

## Figures and Tables

**Table:** Characteristics and treatment response of agitated patients treated with inhaled loxapine in the prehospital setting.

					CGI		PANSS-EC		
							
Patient number*	Age	Gender	Psychiatric diagnosis	Level of agitation	Pre	Post	Pre	Post	Time to response (min.)
1	52	F	BPD	Mild	3	1	17	5	2
2	60	F	SCAD	Moderate	4	1	21	6	4
3	46	F	SCZD	Severe	6	1	30	8	9
6	21	M	SCAD	Mild	3	1	14	5	3
7	47	M	BPD	Moderate	4	1	21	7	5
8	65	M	SCZD	Severe	6	1	31	10	10
9	63	M	BPD	Severe	6	3	28	16	10†‡
10	46	M	BPD	Moderate-severe	5	1	26	6	5
11	33	M	SCZD	Moderate-severe	5	1	24	8	4
12	37	M	SCZD	Moderate-severe	7	4	33	21	10†§

*F*, female; *M*, male; *BPD*, bipolar disorder; *SCAD*, schizoaffective disorder; *SCZD*, schizophrenia; *CGI*, Clinical Global Impression; *PANSS*-*EC*, Positive and Negative Syndrome Scale–Excited Component.

* Patients no. 4 and 5 refused treatment with inhaled loxapine.

† 10 minutes after loxapine administration, 5 mg of intranasal midazolam was also administered to the patient.

‡ Agitation intensity after midazolam CGI = 1, PANSS-EC = 9.

§ Agitation intensity after midazolam CGI = 1, PANSS-EC = 6.
